# Parents of youth who self-injure: a review of the literature and implications for mental health professionals

**DOI:** 10.1186/s13034-015-0066-3

**Published:** 2015-09-28

**Authors:** Alexis E Arbuthnott, Stephen P Lewis

**Affiliations:** University of Guelph, Guelph, ON N1G 2W1 Canada

**Keywords:** Non-suicidal self-injury, Self-harm, Parents, Youth, Review, Mental health

## Abstract

Non-suicidal self-injury (NSSI) is a common mental health concern among youth, and parents can be valuable supports for these youth. However, youth NSSI can have a significant impact on parents’ wellbeing, which may in turn alter parents’ ability to support the youth. To date, no single article has consolidated the research on parents of youth who self-injure. This review synthesizes the literature on parent factors implicated in youth NSSI risk, the role of parents in help-seeking and intervention for youth NSSI, and the impact of youth NSSI on parent wellbeing and parenting. Clinical implications for supporting parents as they support the youth are also discussed, and recommendations for future research are outlined.

## Introduction

Non-suicidal self-injury (NSSI) is the intentional destruction of one’s own body tissue (e.g., cutting, burning) without conscious suicidal intention [1]. NSSI commonly takes the form of cutting, scraping, carving or burning the skin, hitting oneself, or biting oneself [2, 3], though other methods are also reported [4]. Approximately 18% of adolescents have a history of at least one episode of NSSI [5], and over a quarter of these adolescents engage in NSSI repeatedly [6]. Indeed, the average age at NSSI onset is in the early-to-mid teen years [7, 8]. Youth who engage in NSSI are more likely than those who do not self-injure to have at least one diagnosed mental illness (e.g., mood disorders, eating disorders) [9, 10], and to have a history of suicide ideation and suicide attempts [2, 9, 10]. It is common for youth who engage in NSSI to also engage in other maladaptive behaviours such as substance abuse and disordered eating [10–14].

NSSI has emerged as a prominent mental health concern among youth. However, NSSI not only affects the health of youth, it can also have a significant impact on parents’ wellbeing and ability to support their youth [15–17]. To date, no single paper has consolidated the literature on parents of youth who self-injure. A review paper which provides a thorough understanding of the role of parents in youth NSSI may better equip clinicians to treat youth NSSI by involving parents as valuable resources in the youth’s circle of care. Indeed, when parents are appropriately supported, they can be instrumental throughout a young person’s NSSI recovery process [18–20]. Such a review may also help to identify where research is needed to further understand how parent factors play a role in the context of NSSI onset and treatment among youth, and how to equip parents such that they are better able to support their youth. This review begins with a synthesis of the literature examining parents of youth who engage in NSSI, including the risks for NSSI associated with parents, the role of parents during help-seeking and treatment for NSSI, and the impact of youth NSSI on parent wellbeing and ability to support the youth. Next, clinical implications for supporting parents are explored. Finally, gaps in the literature are identified and avenues for further research are suggested.

## Review

**Fig. 1:**
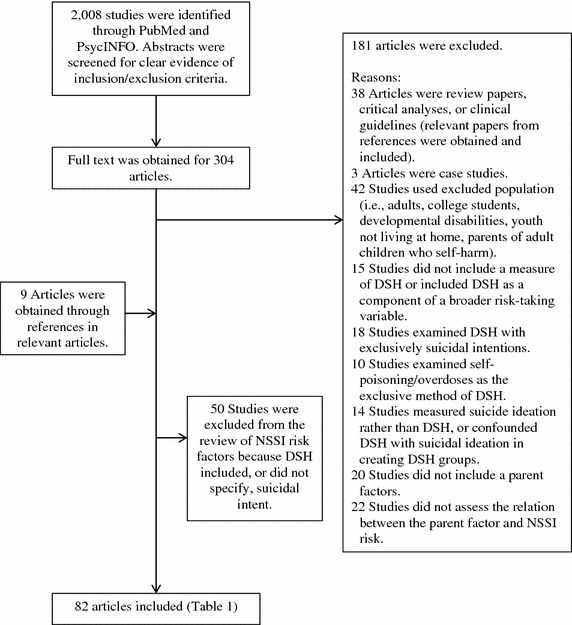
Flow diagram of identified studies.

Papers for this review were identified through the Psych-Info and PubMed databases using the search query (parent* OR family OR interpersonal OR caregiver) AND (self-harm* OR self-injur* OR self-mutilat*) AND (child* OR youth OR adolescen* OR teen OR student OR young). References of resultant papers were also reviewed. Figure 1 outlines the study acquisition and inclusion process. The following inclusion criteria were used: studies had to be peer-reviewed, written in English, and examined NSSI or non-suicidal self-harm among children and/or adolescents (≤19 years). Included studies also had to examine the role of parents in relation to NSSI in at least one of four categories: youth NSSI risk factors; youth help-seeking for NSSI; intervention for youth NSSI; and parent experiences of youth NSSI. Articles were excluded for the following reasons: NSSI or self-harm was examined in young adults or college student populations; samples were drawn from populations with developmental disabilities, psychosis, or youth who were not living at home (e.g., incarcerated youth, street youth); the harm to self was accidental or socially sanctioned (e.g., salt and ice challenges).

**Fig. 2:**
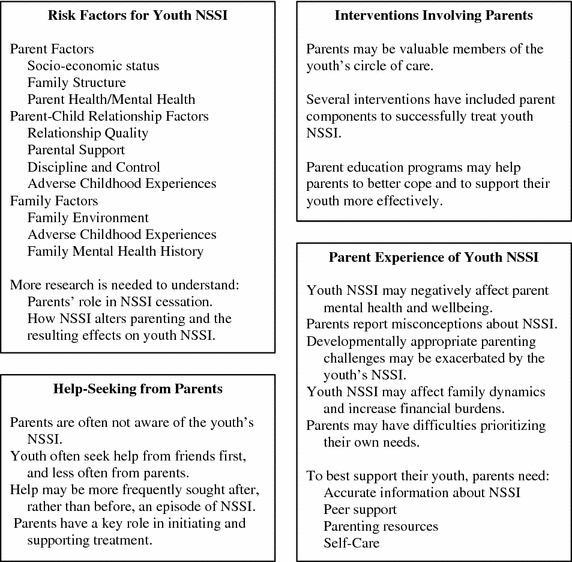
Visual summary of the role of parents in youth NSSI.

Although the initial intent of this review was to examine parents in relation to youth NSSI specifically, the review was expanded to include deliberate self-harm (DSH) in combination with NSSI. DSH encompasses NSSI behaviours as well as behaviours with indirect harm (e.g., self-poisoning, overdoses), and DSH may or may not include behaviours with suicidal intent. Thus, NSSI is subsumed under DSH. The focus was broadened for two reasons. First, there is a paucity of research examining the role of parents during help-seeking and treatment for NSSI specifically, and the authors were unable to locate any peer-reviewed study examining the impact of exclusive NSSI on parent wellbeing. Second, NSSI and DSH are often examined on a continuum of self-harming behaviours rather than as distinct categories [21, 22]. To this end, and for many studies, it was impossible to determine which behaviour (i.e., NSSI versus DSH) was measured based on the methodology provided in the text. Thus, expanding the scope of the review to include DSH as well as NSSI may provide a more comprehensive picture of the role of parents in youth NSSI. The term NSSI is used throughout this review when the study included NSSI behaviours; the reader should note that at times these studies may also have included behaviours that extended beyond the definition of NSSI. To best approximate the goals of the initial review, studies of DSH that clearly did not include NSSI (i.e., self-poisoning was the only method examined; only behaviours with suicidal intent were included; or suicide ideation confounded the measure of self-harm), were excluded. Furthermore, as there may be key differences between adolescents who engage in DSH with suicidal intent versus nonsuicidal intent [23–25], only studies measuring exclusively nonsuicidal DSH were included in the review of risks for NSSI associated with parents. A total of 82 articles^a^ were included in this review (Table 1). A visual summary of the role of parents in youth NSSI that emerged from this review is provided in Fig. 2.

**Table 1:** Studies included in the review of parents’ role in youth NSSI

Risk factors associated with parents^a^		Help-seeking from parents	Interventions involving parents	Impact on parent wellbeing
Cross-sectional	Longitudinal			
*Clinical Sample* ^b^ Adrian et al. [26] Boxer [31] Esposito-Smythers et al. [40] Guertin et al. [43] Kaess et al. [50] Tan et al. [63] Tuisku et al. [67] Venta and Sharp [68] Warzocha et al. [69] Wolff et al. [71] *Cohort Sample* Baetens et al. [29] *Community Sample* Deliberto and Nock [37] Wedig and Nock [70] *School Sample* Baetens et al. [28] Bjärehed and Lundh^c^ [11] Brausch and Gutierrez [23] Brunner et al. [32] Brunner et al. [33] Cerutti et al. [34] Claes et al. [35] Di Pierro et al. [38] Duke et al. [39] Giletta et al. [42] Hargus et al. [45] Hay and Meldrum [46] Kaminski et al. [51] Laye-Gindhu and Schonert-Reichl [54] Liang et al. [56] Lloyd-Richardson et al. [2] Mossige et al. [58] Swahn et al. [61] Taliaferro et al. [62] Tang et al. [64] Yates et al. [72] Zetterqvist et al. [3]	*Clinical Sample* ^b^ Cox et al. [36] Hurtig et al. [47] Jantzer et al. [48] Tuisku et al.^d^ [66] *Cohort Sample* Baetens et al. [30] Geulayov et al. [41] Lereya et al. [55] Page et al. [59] *Community Sample* Hankin and Abela [44] Keenan et al. [52] *School Sample* Andrews et al. [27] Hilt et al. [12] Jutengren et al. [49] Law and Shek [53] Lundh et al. [57] Shek and Yu [60] Tatnell et al. [65] Yates et al. [72] You and Leung [73]	*Qualitative* Berger et al. [80] Rissanen et al. [83] Fortune et al. [82] Fortune et al. [74] *Cross-Sectional* De Leo and Heller [78] Evans et al. [77] Fadum et al. [81] Motjabai and Olfson [76] Rossow and Wichstrøm [75] Watanabe et al. [79]	*Cognitive Behaviour Therapy* Brent et al. [88] Taylor et al. [89] *Dialectical Behaviour Therapy* Fleischhaker et al. [91] Geddes et al. [92] Mehlum et al. [93] Tørmoen et al. [94] Woodberry and Popenoe [95] *Family Based Therapy* Huey et al. [85] Ougrin et al. [86] *Psychodynamic Therapy* Rossouw and Fonagy [87] *Parent Education Program* Pineda and Dadds [96] Power et al. [98] Tambourou et al. [97]	*Qualitative* Byrne et al. [15] McDonald et al. [16] Oldershaw et al. [17] Rissanen et al. [99] Rissanen et al. [20] *Cross-Sectional* Morgan et al. [100]

Samples derived from Australia [16, 27, 65, 78, 80, 92, 96, 97], Belgium [28–30, 35], Canada [54], China [53, 56, 60, 64, 73], England [17, 45, 74, 77, 82, 86, 87, 89], Europe (11 countries sampled for a single study) [33], Finland [20, 47, 66, 67, 83, 99], Germany [32, 48, 50, 91], Ireland [15, 98, 100], Italy [34, 38, 42], Japan [79], Netherlands [35, 42], Norway [58, 75, 81, 93, 94], Poland [69], Singapore [63], Sweden [3, 11, 49, 57], United Kingdom [41, 55, 59, 76] and the United States [2, 12, 23, 26, 31, 36, 37, 39, 40, 42–44, 46, 51, 52, 61, 62, 68, 70–72, 85, 88, 95].

^a^Studies in which nonsuicidal DSH cannot be distinguished from DSH with suicidal intent, (e.g., sample consists of DSH regardless of intent or intent is not specified) are excluded.

^b^Includes inpatient [26, 31, 43, 50, 68, 69, 71] and outpatient [63, 66, 67] youth samples as well as samples of youth with specific diagnoses (i.e., bipolar disorder [40], ADHD [47]), and youth of parents with specific diagnoses (i.e., cancer [48], mood disorders [36]).

^c^Although a test–retest design was used, relevant results were presented for Time 1 and Time 2 cross-sectionally.

^d^Only the first follow-up (1 year after baseline) is included in this review, as the mean age at the second follow-up (8 years after baseline) was beyond the age for inclusion.

### Risks for NSSI associated with parents

Fifty-three studies [2, 3, 11, 12, 23, 26–73] met the inclusion criteria for this section of the review. Table 2 outlines all potential NSSI risk factors associated with parents that have been measured across the included studies. A variety of background factors associated with parents (i.e., socio-economic status, family structure, parent health and mental health history), parent–child relationship factors (i.e., relationship quality, parent support, discipline and control, affect towards parents, adverse childhood experiences associated with parents specifically), and family system factors (i.e., family environment, adverse childhood experiences associated with the family system, family mental health history) have been associated with elevated risk for NSSI. Many background parent factors (e.g., parental level of education, family socioeconomic status, parent marital status, maternal depression) are widely used as covariates in youth NSSI research; as such, it is not unlikely that the authors may have missed some studies that should have been included in this review despite the intensive search and screening process.

**Table 2:** Risk factors for youth NSSI associated with parents

Parent factor	Design	Location	Measures	Summary of findings
*Parent Background Factors*				
Parent Socio-Economic Status				
Education	CS [29, 42, 54, 56, 58]; L [36, 59]	Belgium [29], Canada [54], China [56], Italy [42], Netherlands [42], Norway [58], United Kingdom [59], United States [36, 42]	Researcher Derived Questionnaire [29, 36, 42, 54, 56, 58, 59]	No differences in NSSI risk [36, 42, 54, 56, 58] Elevated risk for NSSI associated with lower parent education level [29] Lower maternal education during pregnancy weakly protected against NSSI risk in adolescence [59]
Unemployment	CS [3, 29, 33, 58]	Belgium [29], Europe [33], Norway [58], Sweden [3]	Researcher Derived Questionnaire [3, 29, 33, 58]	No difference in NSSI risk [58] Elevated risk for NSSI associated with parent unemployment [3, 29, 33]
Lower income	CS [29, 56]; L [36, 59]	Belgium [29], China [56], United Kingdom [59], United States [36]	Researcher Derived Questionnaire [29, 36, 56, 59]	No differences in NSSI risk [36, 56] Elevated risk for NSSI [29, 59]
Financial problems	CS [3, 58]; L [47]	Finland [47], Norway [58], Sweden [3]	Researcher Derived Questionnaire [3, 47, 58]	Elevated risk for NSSI [3, 47, 58] Parents receiving social welfare benefits elevated risk for NSSI [58] Parental ownership of the house they live in was not associated with NSSI risk [58]
Family social status	L [47, 59, 60]	China [60], Finland [47], United Kingdom [59]	Researcher Derived Questionnaire [47, 59, 60]	No differences in NSSI risk [47, 59, 60]
Family Structure				
Non-intact family	CS [2, 3, 29, 32, 33, 45, 46, 50, 51, 54, 56, 58]; L [47]	Belgium [29], Canada [54], China [56], England [45], Europe [33], Finland [47], Germany [32, 50], Norway [58], Sweden [3], United States [2, 46, 51]	Researcher Derived Questionnaire [2, 3, 29, 32, 33, 45–47, 50, 51, 54, 56, 58]	No differences in NSSI risk [2, 29, 45, 46, 50] Elevated risk for NSSI [3, 32, 47, 54, 58] Elevated risk for NSSI associated with not living with biological parent [33] Elevated risk for NSSI associated with youth living with mother or father and a stepparent, or living with neither mother nor father [51] Elevated risk with single-parent family [56]
Parents divorced	CS [45, 67, 69]; L [47, 60]	China [60], England [45], Finland [47, 67], Poland [69]	Researcher Derived Questionnaire [45, 47, 60, 67, 69]	No differences in NSSI risk [45, 67] Elevated risk for NSSI [69] Elevated NSSI risk associated with youth whose parents were divorced and remarried to other people [60] Not meeting with a divorced parent associated with NSSI risk among youth with ADHD [47]
Parent Health and Mental Health History				
Illness or disability	CS [37, 54]; L [48]	Canada [54], Germany [48], United States [37]	Developmental Questionnaire [37]; Inclusion criteria [48]; Researcher Derived Questionnaire [54]	No differences in NSSI risk associated with the number of miscarriages a mother has had [37] Trend toward significant NSSI risk associated with parent history of cancer [48] Elevated risk for NSSI associated with parent history of a serious illness or disability [54]
Mental illness	CS [40]; L [36]	United States [36, 40]	Beck hopelessness Scale [36]; Family History Screen [40]; Hamilton Depression Inventory [36]; Structured Clinical Interview for *DSM*-*IV* [36, 44]; Structured Clinical Interview for the DSM-IV Diagnosis of Personality Disorders [36]	No differences in NSSI risk associated with parental history of mood disorders [40], depression, bipolar disorders, anxiety disorder, posttraumatic stress disorder, or cluster B personality disorder [36] Elevated risk for NSSI associated with lower depressive symptoms among youth of parents with a history of depression [36] Elevated risk for NSSI associated with maternal depression [44]
NSSI/DSH, suicide ideation, suicide attempt	L [36, 41]	United Kingdom [41], United States [36]	Columbia University suicide history form [36]; Life Event Questionnaire [41]; Medical Damage Lethality Scale [36]; Self-Injurious Behavior Scale [36]	No differences in NSSI risk associated with parental history of suicide attempts [36, 41], suicide ideation, or NSSI/DSH [36]
Alcohol and substance abuse	L [36]	United States [36]	Structured Clinical Interview for *DSM*-*IV* [36]	No differences in NSSI risk associated with parental history of alcohol or substance abuse [36]
Parental stress	CS [29]	Belgium [29]	Nijmeegse Vragenlijst voor Opvoedingssituaties [29]	No difference in NSSI risk [29]
Parent Abuse History				
Abuse	L [36]	United States [36]	Childhood Experiences Questionnaire [36]; Abuse Dimensions Inventory [36]; Demographic Questionnaire [36]	No differences in NSSI risk for parent history of physical or sexual abuse [36]
*Parent-Child Relationship Factors*				
Quality of Relationship				
Relationship quality	CS [38]; L [12]	Italy [38], United States [12]	Inventory of Parent and Peer Attachment [12]; Youth Questionnaires [38]	No differences in NSSI risk associated with relationship quality with fathers [38] Elevated risk for NSSI associated with lower overall relationship quality [12], and lower quality relationships with mothers [38] Higher NSSI frequency is associated with lower relationship quality with both mothers and fathers [38] NSSI predicted an increase in positive relationship quality both overall and with fathers [12]
Connectedness with parents	CS [62]	United States [62]	Minnesota Student Survey [62]	Elevated risk for NSSI associated with less connectedness with parents [62]
Attachment and alienation	CS [68]; L [65, 72]	Australia [65], United States [68, 72]	Child Attachment Interview [68]; Adolescent Attachment Questionnaire [65]; Inventory of Parent and Peer Attachment—Alienation subscale [72]	Elevated risk for NSSI onset and maintenance associated with attachment anxiety [65] Individuals who had ceased NSSI continued to have greater attachment anxiety compared to controls, but less than those who maintained NSSI [65] Attachment classification (secure, dismissing, preoccupied, disorganized) did not predict NSSI [68] The indirect path between parental criticism and NSSI risk through parental alienation accounted for much of the direct relation between parental criticism and NSSI youth from high-income families [72]
Support from Parents				
General support	CS [23, 28, 29, 35, 61]	Belgium [28, 29, 35], Netherlands [35], United States [23, 61]	Parent Behavior Scale-Shortened Version (combines items assessing autonomy, positive parenting, reward, and rules) [29]; Child and Adolescent Social Support Scale (Parent Subscale) [23]; Level of Expressed Emotions Scale—Lack of emotional support subscale [28]; Relational Support Inventory [35]; Researcher Developed 5-item scale [61]	No differences in NSSI risk [29] Elevated risk for NSSI associated with lower support from parents [23, 28, 35, 61] Interaction between support and parent behavioural control, such that high control and low support increased the change for NSSI [29] Lack of parental emotional support had a direct effect on NSSI frequency and an indirect effect through depressive symptoms [28] Parent support moderated the relation between bullying/victimization and NSSI, such that bullying/victimization and NSSI are only significantly related at low levels of parental support [35] Parent support moderated the relation between depressed mood and NSSI, such that among participants who engaged in bullying there is a stronger association between depressed mood and NSSI at low levels of parental support [35]
Rule-setting	L [30]	Belgium [30]	Parent Behavior Scale-Shortened Version (rule-setting subscale only) [30]	NSSI predicted less future perceived parental rule-setting among adolescents with high psychological distress [30] Increased rule-setting associated with parent-reported awareness of youth’s NSSI [30]
Positive parenting	L [30]	Belgium [30]	Parent Behavior Scale-Shortened Version (positive parenting subscale only) [30]	No differences in NSSI risk [30]
Criticism	CS [28, 70]; L [72]	Belgium [28], United States [70, 72]	Five Minute Speech Sample [70]; Multidimensional Perfectionism Scale—Parental Criticism subscale [72]; Level of Expressed Emotions Scale—Parental Criticism Subscale [28]	Greater parental criticism associated with an elevated risk for NSSI presence in both boys and girls [70, 72], and with repeated NSSI in boys from high-income families [72] Adolescent self-criticism moderated the relation between parental criticism and NSSI such that adolescent self-criticism was associated with NSSI at borderline and high levels of parental criticism, but not at low levels of parental criticism [70] Parental criticism had only an indirect effect on NSSI frequency through self-criticism [28] An indirect path between parental criticism and NSSI risk through parental alienation accounted for much of the direct relation between parental criticism and NSSI risk among youth from high-income families [72]
Invalidation	CS [63]	Singapore [63]	Invalidating Childhood Environment Scale [63]	Elevated risk for NSSI associated with greater parental invalidation [63]
Expressed emotion	CS [70]	United States [70]	Five Minute Speech Sample [70]	No differences in NSSI risk associate with emotional over-involvement [70] Elevated risk for NSSI associated with greater expressed emotion [70]
Interest, understanding attention	CS [33]; L [47]	Europe [33], Finland [47]	Three items [33]; Self-Report Questionnaire [47]	No differences in NSSI risk associated with parental interest for youth with ADHD [47] Elevated risk for NSSI associated with perception that parents do not pay attention to youth [33], and that parents do not understand the youth’s problems [33] NSSI risk higher for females, related to males, when reporting that parents do not understand youth’s problems [33]
Parental hostility	L [55]	United Kingdom [55]	Researcher-Developed Questionnaire [55]	No differences in NSSI risk [55]
Discipline and Control				
Authoritative parenting	CS [46]	United States [46]	12-Item Scale [46]	Authoritative parenting diminished the negative effects of bullying victimization on NSSI [46]
Behavioural control	CS [29]; L [30]	Belgium [29, 30]	Parent Behavior Scale-Shortened Version (combined punishment, harsh punishing, and neglect subscales [29]; or combined punishment and harsh punishing subscales [30])	No differences in NSSI risk when reported by parents [29] Elevated risk for NSSI associated with greater behavioural control when reported by youth [29] No unique risk in NSSI beyond other parenting variables [30] Interaction between behavioural control and support from parents, such that high control and low support increased the change for NSSI [29]
Harsh parenting	L [49, 52]	Sweden [49], United States [52]	Conflict Tactics Scale-Child Version [52]; Two measures capturing parents’ angry outbursts and coldness-rejection [49]	Elevated risk for NSSI associated with harsher parenting [49] Trend towards elevated risk for NSSI associated with harsher parenting [52] No unique variance in NSSI predicted by harsh parenting when the model included peer victimization, though this was moderated by adolescent’s gender [49]
Psychological control	CS [29]; L [30]	Belgium [29, 30]	Psychological Control Scale [29, 30]	No differences in NSSI risk when reported by parents [29] Elevated risk for NSSI associated with greater psychological control when reported by youth [29] No unique risk for NSSI beyond other parenting variables [30]
Monitoring	CS [61]	United States [61]	Researcher Developed 4-item Scale [61]	Elevated risk for NSSI associated with lower parental monitoring [61]
Emotion socialization	CS [26]	United States [26]	Emotions as a child [26]	Elevated risk for NSSI associate with punishing emotion socialization when combined with other family relational problems, though this risk may be mediated by emotion regulation [26]
Youth Affect Towards Parents				
Idealization of parents	CS [38]	Italy [38]	Youth Questionnaire [38]	Elevated risk for NSSI associated with idealization of mothers but not of father [38]
Feelings towards parents	CS [11]	Sweden [11]	Emotional Tone Index [11]	Elevated risk for NSSI associated with absence of positive feelings, more negative feelings, and overall feelings (more negative and less positive feelings, combined) towards parents [11] No unique variance in NSSI predicted beyond that which was predicted by youth’s rumination/negative thinking ( [11]; Time 1) Unique variance in NSSI predicted beyond that which was predicted by youth’s rumination/negative thinking ([11]; Time 2)
Dysphoric relations	L [57]	Sweden [57]	Researcher-Derived Depression Index subscale [57]	With fatigue, dysphoric relations to parents predicted NSSI [57]
Academic expectations	CS [63]	Singapore [63]	Academic Expectations Stress Inventory [63]	Elevated risk for NSSI associated with greater stress from parental academic expectations [63]
Adverse Childhood Experiences				
Antipathy	CS [50]	Germany [50]	Childhood Experiences of Care and Abuse Questionnaire [50]	Elevated risk for NSSI associated with antipathy from both mothers and fathers [50] Paternal antipathy associated with interpersonal influence functions of NSSI [50]
Maladaptive parenting	L [55]	United Kingdom [55]	Researcher Derived Questionnaire [55]	Parental hitting or shouting in preschool years predicted NSSI in adolescence [55]
Abuse by parent	CS [50, 58, 64]	China [64], Germany [50], Norway [58]	Childhood Experiences of Care and Abuse Questionnaire [50]; Conflict Tactics Scales Parent Child version [64]; Researcher Derived Questionnaire [58]	Elevated risk for NSSI associated with verbal abuse by a parent [58] Elevated risk for NSSI associated with physical abuse by a parent [58, 64], and by fathers specifically [50] Maternal physical abuse predicted peer identification functions of NSSI [50]
Physical neglect	CS [34, 38, 50]	Germany [50], Italy [34, 38]	Boricua Child Interview [38]; Childhood Experiences of Care and Abuse Questionnaire [50]; Life-Stressor Checklist-Revised [34]	No difference in NSSI risk associate with physical neglect [34] Elevated NSSI risk associated with physical neglect from mothers [50] Greater NSSI frequency, but not presence, was associated with physical neglect from a parent [38] Paternal neglect predicted peer identification functions of NSSI [50]
*Family Systems Factors*				
Family Environment				
Family functioning	CS [29, 43]; L [53]	Belgium [29], China [53], United States [43]	Chinese Family Assessment Inventory [53]; McMaster Family Assessment Device—General Functioning Subscale [43]; Vragenlijst Gezinsproblemen [29]	No differences in NSSI risk when reported by youth [43], or parents [29] Elevated risk for NSSI associated with lower family functioning [53]
Support	CS [67, 69, 71]; L [27, 65, 66]	Australia [27, 65], Finland [66, 67], Poland [69], United States [71]	Multidimensional Scale of Perceived Social Support [27, 65]; Perceived Social Support Scale-Revised [66, 67]; Researcher Derived Questionnaire [69]; Survey of Children’s Social Support [71]	No differences in NSSI risk [66, 69] Elevated risk for NSSI presence [27, 67, 71], onset and maintenance associated with lower support from parents [65] NSSI onset associated with a decrease in family support [65] NSSI cessation associated with an increase in family support over time, though individuals who had ceased NSSI continued to perceive lower levels of support from family relative to individuals with no NSSI history [65]
Adaptability and cohesion	CS [26, 51, 56], L [36]	China [56], United States [26, 36, 51]	Family Environment Scale [26]; Family Adaptability and Cohesion Evaluation Scale-II [36]; Family Cohesion and Adaptability Scale-Chinese Version [56]; Vaux Social Support Record [51]	No differences in NSSI risk associated with family adaptability [36] Elevated risk for NSSI associated with greater family rigidity [56] Elevated risk for NSSI associated with lower family cohesion [26, 51, 56], though this risk may be mediated by emotion regulation [26] Elevated NSSI risk associated with lower family adaptability and cohesion among youth of parents with a history of depression [36]
Conflict	CS [26]	United States [26]	Family Environment Scale [26]	Elevated risk for NSSI associated with greater family conflict, though this risk may be mediated by emotion regulation [26]
Invalidation	L [73]	China [73]	Family Invalidation Scale [73]	Elevated risk for NSSI [73]
Arguments between parents	CS [45]	England [45]	Self-Report Questionnaire [45]	No difference in NSSI risk [45]
Loneliness	CS [42]	Italy [42], Netherlands [42], United States [42]	Social and Emotional Loneliness Scale for Adults-Adapted [42]	Elevated risk for NSSI associated with family-related loneliness among Dutch and US adolescents, but not among Italian adolescent [42] Elevated risk for repeated NSSI associated with family-related loneliness [42]
Socializing with family	L [47]	Finland [47]	Self-Report Questionnaire [47]	Elevated risk for NSSI associated with youth with ADHD who socialize less with the family [47]
Adverse Childhood Experiences				
Domestic violence	CS [34, 39, 58, 62], L [55]	Italy [34, 38], Norway [58], United Kingdom [55], United States [39, 62]	Life-Stressor Checklist-Revised [34]; Minnesota Student Survey [39]; Research Derived Questionnaire [55, 58]; Minnesota Student Survey [62]	No difference in NSSI risk associated with witnessing family violence [39, 62] Elevated risk for NSSI associated with witnessing family violence [34] Elevated risk for NSSI associated with domestic violence in preschool years [55], and with witnessing parents being verbally or physically abused [58]
Abuse	CS [39, 69]	Poland [69], United States [39]	Minnesota Student Survey [39]; Researcher Derived Questionnaire [69]	No differences in NSSI risk associated with sexual abuse in the family [69] Elevated risk for NSSI associated with both physical and sexual abuse by a household adult [39]
Negative life events in the family	CS [29]	Belgium [29]	Summation of 19 events (e.g., financial problems, death in the family) [29]	No differences in NSSI risk when reported by parents [29]
Death of a family member	CS [45, 69]	England [45], Poland [69]	Researcher Derived Questionnaire [45, 69]	No difference in NSSI risk [45, 69]
Family Health and Mental Health History				
Health problems	CS [32]	Germany [32]	Researcher Derived Questionnaire [32]	Elevated risk for occasional, but not repetitive, NSSI associated with some (but not many) health problems in the family [32]
Mental illness	CS [31, 37, 69]	Poland [69], United States [31, 37]	Personal and Family History Questionnaire [37]; Review of medical records [31]; Researcher Derived Questionnaire [69]	No differences in NSSI risk associated with a family history of mental illness [31, 69], emotional or behavioural problems, depression, bipolar disorder, anxiety, eating disorder, schizophrenia, or Tourette’s [37]
NSSI/DSH or suicide ideation	CS [37, 45]	England [45], United States [37]	Personal and Family History Questionnaire [37]; Self-Report Questionnaire [45]	No differences in NSSI risk associated with a family history of NSSI/DSH [37, 45] Elevated risk for NSSI associated with a family history of suicide ideation [37]
Alcohol and substance abuse	CS [37, 39, 62, 69]	Poland [69], United States [37, 39, 62]	Minnesota Student Survey [39]; Personal and Family History Questionnaire [37]; Population Based Survey [62]; Researcher Derived Questionnaire [69]	No differences in NSSI risk associated with a family history of alcohol [69] or substance [62] abuse Elevated risk for NSSI associated with a family history of alcohol or substance abuse [37] Elevated risk for NSSI when alcohol or substance use caused problem [39]
Criminality or violence	CS [31, 37]	United States [31, 37]	Personal and Family History Questionnaire [37]; Review of medical records [31]	Elevated risk for NSSI associated with both criminality [31] and violence [31, 37]

*CS* cross-sectional and *L* longitudinal.

Research examining youth NSSI risk beyond the use of correlations and group differences is still in its infancy. Cross-sectional research methods make it difficult to determine the direction of the effect (i.e., whether the parent factor influences youth NSSI, whether youth NSSI changes parent behaviour, or some combination). Although an increasing number of longitudinal studies have used factors associated with parents to predict NSSI risk (see Table 1), only three studies [12, 30, 65] have examined the associations between NSSI and future parent variables, regardless of parents’ awareness of the youth’s NSSI. Similarly, more research is needed to examine the full course of youth NSSI—including NSSI cessation—in relation to factors associated with parents; despite the role that parents and families have in treatment for youth NSSI, only one study in this review examined family factors in NSSI cessation [65]. Understanding the role of parents over the course of NSSI may allow clinicians to better equip parents to support their youth. Although there is no standard model for how parents and adolescents should interact to reduce risk for NSSI, some parental responses towards adolescent emotions (e.g., comfort, validation, support) may protect against NSSI [35] or may encourage NSSI cessation [65]. Thus, equipping parents with the skills necessary to model adaptive emotional acceptance, regulation and expression may be helpful in enhancing parents’ ability to support their youth.

### Help-seeking and parents

Many youth who engage in NSSI tell no one about it [74, 75], and reported parental awareness rates of youth NSSI are considerably lower than actual youth NSSI rates [30, 76]. Those adolescents who seek help most frequently do so from peers and less frequently from family members, including parents [74, 75, 77–79]. One study found that youth with a history of NSSI were less likely to know how parents could help, more likely to suggest that nothing could be done by parents, and less likely to suggest that parents talk to youth who self-injure or that parents refer these youth to professional help [80].

Help from family may more frequently be sought after, rather than before, an episode of NSSI [74, 77], and has been associated with subsequent help-seeking from health services [81]. Youth may be more likely to seek help from parents when they feel as though their parents authentically care for them, and they are able to openly discuss self-injury with their parents [82, 83]. This highlights the need for clinicians who work with families in which a youth self-injures to foster open communication about emotions in family contexts early in the treatment process. Disclosure of NSSI is sometimes made to parents on behalf of the youth by school personnel or a physician [17], and parents who receive poor initial support from schools and health professionals may be unlikely to continue to seek help [17]. The period of initial NSSI discovery may represent a key opportunity for parents to gain knowledge about NSSI, and to encourage professional help-seeking for their youth when warranted.

### Interventions involving parents

Parents may have an essential role in initiating and supporting treatments for youth NSSI [20, 81, 84], Youth may be more likely to accept professional help for NSSI when parents are supportive of treatment [20]. For example, parents’ expectations about the helpfulness of counseling may influence the youth’s decision to attend—or not attend—counseling sessions following presentation at an emergency department following NSSI [84]. A caring environment and open discussion about NSSI may contribute not only to help seeking [83], but also toward supporting the youth to understand, work through, and stop NSSI [20].

Only a handful of studies have examined interventions involving parents for NSSI behaviours specifically (i.e., measured as an outcome either in the absence of, or in combination with, DSH with suicidal intent). Studies of family-based therapies included multi-systemic therapy [85] and single-family therapeutic assessments [86]. Although attachment-based family therapy and family-based problem solving have some evidence of being efficacious for suicidal behaviours, outcomes related to NSSI have not yet been investigated [18, 19]. Mentalization-based treatment, which consists of both individual and family psychodynamic psychotherapy, has been examined in relation to NSSI in one study [87]. Studies assessing cognitive behaviour therapies (CBT) for youth NSSI have involved parents through family CBT in addition to individual CBT for the youth [88], or through a parent psycho-education component [89]; the inclusion of family problem solving sessions or parent training in CBT has not yet been assessed in relation to NSSI specifically [18]. Finally, dialectical behaviour therapy for adolescents [90] has gained recent empirical interest for youth NSSI [91–95]; this intervention consists of individual therapy for adolescents, family therapy as warranted, and a multifamily skills training group.

Reviews [18, 19] of interventions for youth DSH, including NSSI, have found that the inclusion of strong parent components in some interventions may result in significant reductions in youth DSH. However, an examination of the efficacy of these treatments is beyond the scope of this review; readers are referred to these review papers [18, 19] for treatment efficacy. Although few studies have assessed the benefits of these interventions on parents’ wellbeing and ability to support their youth, preliminary evidence suggests that parent [95] and family [96] functioning may significantly improve through participation even when youth NSSI behaviours may not [95].

Beyond interventions for youth specifically, parent education programs may have merit in assisting parents to cope with their youth’s NSSI and better support their youth. For example, a school-based program for parents [97] was found to reduce youth NSSI among students of parents who participated; this program consisted of parent education groups that empowered parents to assist each other to improve communication and relationships with youth. Similarly, two support programs (i.e., Resourceful Adolescent Parent Program (RAP-P); [96]; Supporting Parents and Carers (SPACE); [98]) have been reported for parents of youth who have engaged in, or expressed thoughts of, suicidal behaviour or DSH (including NSSI); RAP-P used a single-family format [96], whereas SPACE had a group format [98]. Both programs provided parents with information pertaining to DSH and NSSI in youth, parenting adolescents, and family communication and conflict. SPACE also provided explicit information about parental self-care. When combined with routine care, RAP-P resulted in significant improvements in family functioning. Similarly, parents in the SPACE pilot study reported subsequent decreased psychological distress and greater parental satisfaction. Parents and youth also reported that youth experienced fewer difficulties following parent participation [96, 98]. Taken together, parent participation in interventions pertaining to youth NSSI may have positive outcomes both for the youth and parent.

### Impact on parent wellbeing

The process of supporting a youth who self-injures can be traumatic and emotionally taxing on parents [15–17, 20]. Parents report an abundance of negative emotions (e.g., sadness, shame, embarrassment, shock, disappointment, self-blame, anger, frustration) in relation to their youth’s NSSI [15–17]. Many parents have expressed feeling overwhelmingly alone, isolated and helpless [15–17]. These feelings can be exacerbated by the stigma surrounding NSSI and the perceived absence of services and supports for NSSI [15]. Parents have reported being unable to talk to anyone about the youth’s NSSI or being extremely selective in choosing to whom they disclose (e.g., disclosing to a close friend, but not to family members) [15]. Many parents have reported a desire for peer support from other parents of youth who self-injure [15, 20], with the anticipated benefits involving the sharing of similar circumstances, learning from each other, and relief from knowing that they are not alone [15].

Although parents may recognize that NSSI serves a function for the youth (e.g., to provide relief from distress), many parents have reported being unable to understanding NSSI as chosen behaviour [17, 99]. Indeed, many parents believe common misconceptions about this behaviour [15, 17, 99]. For example, one study assessing parent conceptions about NSSI found that many parents believed that cutting oneself—one of the more common methods of NSSI among youth who self-injure [2, 3]—is a typical phase of adolescence, occurs only in females, is synonymous with a suicide attempt, or is an indicator of a psychological disorder [99]. The availability of accurate information about NSSI has been identified as a priority by parents of youth who self-injure [15].

Youth NSSI may increase parenting burden and stress [17], and parents often report a loss of parenting confidence [15, 16]. Indeed, in families in which a youth self-injures, poor parental wellbeing has been predicted by poor family communication, low parenting satisfaction, and more difficulties for the youth [100]. Although a key developmental process during adolescence is to individuate from parents, many parents report believing their youth was more mature and capable than they really were [99], and many struggled to find and allow the youth an appropriate level of independence [16]. Nervousness about triggering NSSI (i.e., causing an episode of NSSI) can affect parents’ ability to set limits and maintain boundaries [17]. Parents have also reported that typical difficulties associated with parenting adolescents (e.g., bullying, peer pressure, monitoring Internet use) may be intensified when their youth self-injures, as the adolescent’s experiences in these domains may precipitate or maintain NSSI behaviours [15]. Indeed, parents of youth with NSSI have expressed a need for more effective parenting skills [15]. Despite the difficulties associated with NSSI, many parents hope to rebuild a positive relationship with the youth, recognize the importance of parent–child communication in the youth’s wellbeing, and want to help the youth develop emotion regulation and coping strategies [15].

Finally, parents may also experience difficulties balancing and meeting the varying needs of individual family members [15–17]. Disruptions in family dynamics may occur, and the youth with NSSI may be perceived to hold the central position of power within the family [15]. Some parents have reported that caring for the youth who self-harms led to changes in employment (e.g., reducing hours, leaving paid employment), which may have increased financial strain on families [16]. Finally, parents may deny their own needs, and change or limit their lifestyle to increase support for the youth who self-harms [17]. Taken together, youth NSSI and parent factors associated with NSSI risk may be bidirectional; NSSI can have a significant impact on parent wellbeing and parenting, which may in turn affect parents’ ability to support their youth. Accordingly, parents of youth who self-injure may benefit from additional support for themselves as they support their youth.

### Clinical implications for supporting parents

Parents may be valuable members of the youth’s circle of care. One study found that among youth who presented to an emergency department for self-harm, ongoing parental concern was a better predictor of future DSH than clinical risk assessments [101]; thus, under some circumstances, parents may be in a position to gauge their youth’s ongoing wellbeing and alert health professionals about concerns when warranted [99, 101]. Indeed, another study found that many parents consider themselves to be the youth’s principal helper and advocate [20], which may have both positive and negative implications for both parent and youth wellbeing. For many parents, taking care of themselves while their youth struggles with NSSI is challenging [20, 98]. Thus, parents may need to be encouraged to practice self-care [98]. As parents may also benefit from receiving accurate information about NSSI, parenting skills, and social support [15], the inclusion of parents in empirically-informed treatments—such as those listed above—may be an optimal way to provide parents with education, skills training, and peer support that they can draw upon when supporting their youth at home. Parent education programs for parents of youth who self-injure may also have merit and should be investigated in future research.

The Internet may be a unique medium to support parents of youth who self-injure. Researchers have found that parents use the Internet to access both information related to their children’s medical conditions [102–105], and social support that is not being accessed offline [102, 106]. The Internet has the potential to be a particularly effective method to educate parents about more stigmatized mental health issues such as NSSI, and to equip parents to support their youth with these difficulties. Unfortunately, there is an abundance of non-credible and low-quality information about NSSI on the Internet [107]. Thus, clinicians need to be mindful of parents’ use of the Internet to access support for youth NSSI, and be prepared to recommend credible websites containing accurate NSSI information. Mental health professionals may find that the Self-Injury Outreach and Support [108] and Cornell Research Program on Self-Injury and Recovery [109] websites are particularly useful online resource for parents, as they provide credible and accurate information for parents seeking to understand their youth’s NSSI and how to support their youth (e.g., how to talk to their youth about NSSI, treatments for youth NSSI), as well as providing suggestions for additional online and offline resources specific to parents.

### Implications for further research

There are several limitations in the cited studies that suggest avenues for future research. First, there is a paucity of research pertaining to parents of youth who engage in NSSI specifically; much of what is known about these parents is inferred from studies assessing parents of youth who engage in similar behaviours such as self-harm, which may or may not include a suicidal intent. Thus, more research is needed to determine to what extent parents of youth with NSSI differ from parents of youth who self-harm. This information may assist mental health professionals to develop empirically-informed programs for parents of youth who self-injure that may be modeled on programs already existing for parents of youth who self-harm [96, 98].

Next, studies linking parenting factors to NSSI risk are predominantly correlational, and thus causation cannot be inferred. Researchers should consider complex ways in which factors associated with parents might interact to increase risk for, or protect against, NSSI. Similarly, factors that may mediate or moderate the relation between youth NSSI and the effects of this NSSI on parents are not yet known. To date, studies examining the impact of youth NSSI on parent wellbeing and parenting have been almost exclusively qualitatively. Empirical studies are needed in this area to better understand the effects of youth NSSI on parenting and parents’ subsequent ability to support the youth.

Finally, the effects of parent and youth gender on NSSI risks and NSSI impact on parents are unclear. The impact of NSSI on parent wellbeing has almost exclusively been examined through mothers due to an inability to recruit adequate numbers of fathers; thus, these findings should be generalized cautiously to fathers and other caregivers. Similarly, there may be gender differences in NSSI risk and protective factors. For example, connectedness with parents may be particularly important in protecting adolescent females against NSSI [62], and parent–child relationship quality may confer different risks for NSSI when associated with mothers versus fathers [38]. Further research is needed to identify whether fathers have similar experiences to mothers in supporting youth who self-injure, and how factors associated with mothers and fathers may confer different risks or protection for youth NSSI.

## Conclusions

Parents can play a key role in supporting youth who self-injure. However, youth NSSI affects parents’ wellbeing, which may, in turn, affect how parents can support their youth. Providing parents with accurate information about NSSI, parenting skills, and social support may help parents to better support their youth. When working with youth who self-injure, professionals should consider family dynamics and related contextual factors when selecting appropriate interventions for youth; parents may be valuable members of the circle of care. More research is needed to identify salient parent factors affecting youth NSSI risk and parent wellbeing, and to determine the most effective ways to support parents of youth who self-injure. Efforts in this regard may bolster the quality of clinical care provided to youth who self-injure.

## Endnote

^a^A full table outlining the sample, methods, measures, and results for each study is available from the authors upon request.

## Abbreviations

CBT: cognitive behaviour therapy
DSH: deliberate self-harm
NSSI: non-suicidal self-injury
RAP-P: Resourceful Adolescent Parent Program
SPACE: Supporting Parents and Carers

## References

[1] Nock MK, Favazza AR, Nock MK (2009). Nonsuicidal self-injury: definition and classification. Understanding nonsuicidal self-injury: origins, assessment, and treatment.

[2] Lloyd-Richardson EE, Perrine N, Dierker L, Kelly ML (2007). Characteristics and functions of non-suicidal self-injury in a community sample of adolescents. Psychol Med.

[3] Zetterqvist M, Lundh L, Dahlstrom O, Svedin CG (2013). Prevalence and function of non-suicidal self-injury (NSSI) in a community sample of adolescents, using suggested DSM-5 criteria for a potential NSSI disorder. J Abnorm Child Psychol.

[4] Gratz KL, Chapman AL (2009). Freedom from self-harm: overcoming self-injury with skills from DBT and Other treatments.

[5] Muehlenkamp JJ, Claes L, Havertape L, Plener PL (2012). International prevalence of adolescent non-suicidal self-injury and deliberate self-harm. Child Adolesc Psychiatry Ment Health.

[6] Jacobson CM, Gould M (2007). The epidemiology and phenomenology of nonsuicidal self-injurious behavior among adolescents: a critical review of the literature. Arch Suicide Res.

[7] Rodham K, Hawton K, Nock MK (2009). Epidemiology and phenomenology of nonsuicidal self-injury. Understanding nonsuicidal self-injury: origins, assessment, and treatment.

[8] Whitlock J, Eckenrode J, Silverman D (2006). Self-injurious behaviors in a college population. Pediatr..

[9] Janis IB, Nock MK (2009). Are self-injurers impulsive?: results from two behavioral laboratory studies. Psychiatry Res.

[10] Nock MK, Joiner TE, Gordon KH, Lloyd-Richardson E, Prinstein MJ (2006). Non-suicidal self-injury among adolescents: diagnostic correlates and relation to suicide attempts. Psychiatry Res.

[11] Bjärehed J, Lundh LG (2008). Deliberate self-harm in 14-year-old adolescents: how frequent is it, and how is it associated with psychopathology, relationship variables, and styles of emotional regulation?. Cogn Behav Ther.

[12] Hilt LM, Nock MK, Lloyd-Richardson EE, Prinstein MJ (2008). Longitudinal study of nonsuicidal self-injury among young adolescents: rates, correlates, and preliminary test of an interpersonal model. J Early Adolesc.

[13] Laukkanen E, Rissanen ML, Honkalampi K, Kylma J, Tolmunen T, Hintikka J (2009). The prevalence of self-cutting and other self-harm among 13- to 18-year-old Finnish adolescents. Soc Psychiatry Psychiatry Epidemiol.

[14] Ross S, Heath NL, Toste JR (2009). Non-suicidal self-injury and eating pathology in high school students. Am J Orthopsychiatry.

[15] Byrne S, Morgan S, Fitzpatrick C, Boylan C, Crowley S, Gahan H (2008). Deliberate self-harm in children and adolescents: a qualitative study exploring the needs of parents and carers. Clin Child Psychol Psychiatry.

[16] McDonald G, O’Brien L, Jackson D (2007). Guilt and shame: experiences of parents of self-harming adolescents. J Child Health Care..

[17] Oldershaw A, Richards C, Simic M, Schmidt U (2008). Parents’ perspectives on adolescent self-harm: qualitative Study. Br J Psychiatry.

[18] Glenn CR, Franklin JC, Nock MK (2015). Evidence-based psychosocial treatments for self-injurious thoughts and behaviors in youth. J Clin Child Adolesc Psychol.

[19] Ougrin D, Tranah T, Stahl D, Moran P, Asarnow JR (2015). Therapeutic interventions for suicide attempts and self-harm in adolescents: systematic review and meta-analysis. J Am Acad Child Adolesc Psychiatry.

[20] Rissanen ML, Kylma J, Laukkanen E (2009). Helping adolescents who self-mutilate: parental descriptions. J Clin Nurs.

[21] Hooley JM (2008). Self-harming behavior: introduction to the special series on non-suicidal self-injury and suicide. Appl Prev Psychol.

[22] Latimer S, Covic T, Tennant A (2012). Co-calibration of deliberate self harm (DSH) behaviours: towards a common measurement metric. Psychiatry Res.

[23] Brausch AM, Gutierrez PM (2010). Differences in non-suicidal self-injury and suicide attempts in adolescents. J Youth Adolesc.

[24] Hamza CA, Stewart SL, Willoughby T (2012). Examining the link between nonsuicidal self-injury and suicidal behavior: a review of the literature and an integrated model. Clin Psychol Rev.

[25] Ougrin D, Zundel T, Kyriakopoulos M, Banarsee R, Stahl D, Taylor E (2012). Adolescents with suicidal and nonsuicidal self-harm: clinical characteristics and response to therapeutic assessment. Psychol Assess.

[26] Adrian M, Zeman J, Erdley C, Lisa L, Sim L (2011). Emotion dysregulation and interpersonal difficulties as risk factors for nonsuicidal self-injury in adolescent girls. J Abnorm Child Psychol.

[27] Andrews T, Martin G, Hasking P, Page A (2014). Predictors of onset for non-suicidal self-injury within a school-based sample of adolescents. Prev Sci.

[28] Baetens I, Claes L, Hasking P, Smits D, Grietens H, Onghena P (2015). The relationship between parental expressed emotions and non-suicidal self-injury: the mediating roles of self-criticism and depression. J Child Fam Stud.

[29] Baetens I, Claes L, Martin G, Onghena P, Grietens H, Van Leeuwen K (2014). Is nonsuicidal self-injury associated with parenting and family factors?. J Early Adolesc.

[30] Baetens I, Claes L, Onghena P, Grietens H, Van Leeuwen K, Pieters C (2014). Non-suicidal self-injury in adolescence: a longitudinal study of the relationship between NSSI, psychological distress and perceived parenting. J Adolesc.

[31] Boxer P (2010). Variations in risk and treatment factors among adolescents in different types of deliberate self-harm in an inpatient sample. J Clin Child Adolesc Psychol.

[32] Brunner R, Parzer P, Haffner J, Steen R, Roos J, Klett M (2007). Prevalence and psychological correlates of occasional and repetitive deliberate self-harm in adolescents. Arch Pediatr Adolesc Med.

[33] Brunner R, Kaess M, Parzer P, Fischer G, Carli V, Hoven CW (2014). Life-time prevalence and psychosocial correlates of adolescent direct self-injurious behavior: a comparative study of findings in 11 European countries. J Child Psychol Psychiatry.

[34] Cerutti R, Manca M, Presaghi F, Gratz KL (2011). Prevalence and clinical correlates of deliberate self-harm among a community sample of Italian adolescents. J Adolesc.

[35] Claes L, Luyckx K, Baetens I, Van de Ven M, Witteman C (2015). Bullying and victimization, depressive mood, and non-suicidal self-injury in adolescents: the moderating role of parental support. J Child Fam Stud.

[36] Cox LJ, Stanley BH, Melhem NM, Oquendo MA, Birmaher B, Burke A (2012). A longitudinal study of nonsuicidal self-injury in offspring at high risk for mood disorder. J Clin Psychiatry.

[37] Deliberto TL, Nock MK (2008). An exploratory study of correlates, onset, and offset of non-suicidal self-injury. Arch Suicide Res.

[38] Di Pierro R, Sarno I, Perego S, Gallucci M, Madeddu F (2012). Adolescent nonsuicidal self-injury: the effects of personality traits, family relationships and maltreatment on the presence and severity of behaviours. Eur Child Adolesc Psychiatry.

[39] Duke N, Pettingell SL, McMorris BJ, Borowsky IW (2010). Adolescent violence perpetration: associations with multiple types of adverse childhood experiences. Pediatrics.

[40] Esposito-Smythers C, Goldstein T, Birmaher B, Goldstein B, Hunt J, Ryan N (2010). Clinical and psychosocial correlates of non-suicidal self-injury within a sample of children and adolescents with bipolar disorder. J Affect Disord.

[41] Geulayov G, Metcalfe C, Heron J, Kidger J, Gunnell D (2014). Parental suicide attempt and offspring self-harm and suicidal thoughts: results from the Avon longitudinal study of parents and children (ALSPAC) birth cohort. J Am Acad Child Adolesc Psychiatry.

[42] Giletta M, Scholte RHJ, Engels RCME, Ciairano S, Prinstein MJ (2012). Adolescent non-suicidal self-injury: a cross-national study of community samples from Italy, the Netherlands and the United States. Psychiatry Res.

[43] Guertin T, Lloyd-Richardson E, Spirito A, Donalson D, Boergers J (2001). Self-mutilative behavior in adolescents who attempt suicide by overdose. J Am Acad Child Adolesc Psychiatry.

[44] Hankin BL, Abela JRZ (2011). Nonsuicidal self-injury in adolescence: prospective rates and risk factors in a 2-1/2 year longitudinal study. Psychiatry Res.

[45] Hargus E, Hawton K, Rodham K (2009). Distinguishing between subgroups of adolescents who self-harm. Suicide Life Threat Behav.

[46] Hay C, Meldrum R (2010). Bullying victimization and adolescent self-harm: testing hypotheses from general strain theory. J Youth Adolesc.

[47] Hurtig T, Taanila A, Moilanen I, Nordström T, Ebeling H (2012). Suicidal and self-harm behaviour associated with adolescent attention deficit hyperactivity disorder—A study in the Northern Final Birth Cohort 1986. Nord J Psychiatry..

[48] Jantzer V, Groß J, Stute F, Parzer P, Brunner R, Willig K (2013). Risk behaviors and externalizing behaviors in adolescents dealing with parental cancer—a controlled longitudinal study. Psycho-Oncol.

[49] Jutengren G, Kerr M, Stattin H (2011). Adolescents’ deliberate self-harm, interpersonal stress, and the moderating effect of self-regulation: a two-wave longitudinal analysis. J School Psychol.

[50] Kaess M, Parzer P, Mattern M, Plener PL, Bifulco A, Resch F (2013). Adverse childhood experiences and their impact on frequency, severity, and the individual function of nonsuicidal self-injury in youth. Psychiatry Res.

[51] Kaminiski JW, Puddy RW, Hall DM, Cashman SY, Crosby AE, Ortega LAG (2010). The relative influence of different domains of social connectedness on self-directed violence in adolescence. J Youth Adolesc.

[52] Keenen K, Hipwell AE, Stepp SD, Wroblewski K (2014). Testing an equifinality model of nonsuicidal self-injury among early adolescent girls. Dev Psychopathol.

[53] Law BMF, Shek DTL (2013). Self-harm and suicidal attempts among young Chinese adolescents in Hong Kong: prevalence, correlates, and changes. J Pediatr Adolesc Gynecol.

[54] Laye-Gindhu A, Schonert-Reichl KA (2005). Nonsuicidal self-harm among community adolescents: understanding the “whats” and “whys” of self-harm. J Youth Adolesc.

[55] Lereya ST, Winsper C, Heron J, Lewis G, Gunnell D, Fisher HL (2013). Being bullied during childhood and the prospective pathways to self-harm in late adolescence. J Am Acad Child Adolesc Psychiatry.

[56] Liang S, Yan J, Zhang T, Zhu C, Situ M, Du N (2014). Differences between non-suicidal self injury and suicide attempt in Chinese adolescents. Asian J Psychiatry..

[57] Lundh LG, Wångy-Lundh M, Paaske M, Ingesson S, Bjärehed J (2011). Depressive symptoms and deliberate self-harm in a community sample of adolescents: a prospective study. Depress Res Treat.

[58] Mossige S, Huang L, Straiton M, Roen K (2014). Suicidal ideation and self-harm among youths in Norway: associations with verbal, physical and sexual abuse. Child Fam Social Work..

[59] Page A, Lewis G, Kidger J, Heron J, Chittleborough C, Evans J (2014). Parental socio-economic position during childhood as a determinant of self-harm in adolescence. Soc Psychiatry Psychiatr Epidemiol.

[60] Shek DTL, Yu L (2012). Self-harm and suicidal behaviors in Hong Kong adolescents: prevalence and psychosocial correlates. Sci World J.

[61] Swahn MH, Ali B, Bossarte RM, Van Dulmen M, Crosby A, Jones AC (2012). Self-harm and suicide attempts among high-risk, urban youth in the US: shared and unique risk and protective factors. In J Environ Res. Public Health.

[62] Taliaferro LA, Muehlenkamp JJ, Borowsky IW, McMorris BJ, Kugler KC (2012). Factors distinguishing youth who report self-injurious behavior: a population-based sample. Acad Pediatr.

[63] Tan ACY, Rehfuss MC, Suarez EC, Parks-Savage A (2014). Nonsuicidal self-injury in an adolescent population in Singapore. Clin Child Psychol Psychiatry..

[64] Tang CS, Wong WCW, Leung PMS, Chen W, Lee A, Ling DC (2010). Health compromising behaviors among Chinese adolescents: role of physical abuse, school experience, and social support. J Health Psychol..

[65] Tatnell R, Kelada L, Hasking P, Martin G (2014). Longitudinal analysis of adolescent NSSI: the role of intrapersonal and interpersonal factors. J Abnorm Child Psychol.

[66] Tuisku V, Kiviruusu O, Pelkonen M, Karlsson L, Strandholm T, Marttunen M (2014). Depressed adolescents as young adults—Predictors of suicide attempts and non-suicidal self-injury during an 8-year follow-up. J Affect Disord.

[67] Tuisku V, Pelkonen M, Kiviruusu O, Karlsson L, Ruuttu T, Marttunen M (2009). Factors associated with deliberate self-harm behaviour among depressed outpatients. J Adolesc.

[68] Venta A, Sharp C (2014). Attachment organization in suicide prevention research: preliminary findings and future directions in a sample of inpatient adolescents. Crisis.

[69] Warzocha D, Pawełczyk T, Gmitrowicz A (2010). Associations between deliberate self-harm episodes in psychiatrically hospitalized youth and the type of mental disorders and selected environmental factors. Arch Psychiatry Psychother.

[70] Wedig MM, Nock MK (2007). Parental expressed emotion and adolescent self-injury. J Am Acad Child Adolesc Psychiatry.

[71] Wolff JC, Frazier EA, Esposito-Smythers C, Becker SJ, Burke TA, Cataldo A (2014). Negative cognitive style and perceived social support mediate the relationship between aggression and NSSI in hospitalized adolescents. J Adolesc.

[72] Yates TM, Tracy AJ, Luthar SS (2008). Nonsuicidal self-injury among “privileged” youths: longitudinal and cross-sectional approaches to developmental process. J Consult Clin Psychol.

[73] You J, Leung F (2012). The role of depressive symptoms, family invalidation and behavioral impulsivity in the occurrence and repetition of non-suicidal self-injury in Chinese adolescents: a 2-year follow-up study. J Adolesc..

[74] Fortune S, Sinclair J, Hawton K (2008). Help-seeking before and after episodes of self-harm: a descriptive study in school pupils in England. BMC Public Health..

[75] Rossow I, Wichstrøm L (2010). Receipt of help after deliberate self-harm among adolescents: changes over an eight-year period. Psychiatr Serv.

[76] Mojtabai R, Olfson M (2008). Parental detection of youth’s self-harm behavior. Suicide Life Threat Behav.

[77] Evans E, Hawton K, Rodham K (2005). In what ways are adolescents who engage in self-harm or experience thoughts of self-harm different in terms of help-seeking, communication and coping strategies?. J Adolesc.

[78] De Leo D, Heller TS (2004). Who are the kids who self-harm? An Australian self-report school survey. Med J Aust.

[79] Watanabe N, Nishida A, Shimodera S, Inoue K, Oshima N, Sasaki T (2012). Help-seeking behavior among Japanese school students who self-harm: results from a self-report survey of 18,104 adolescents. Neuropsychiatr Dis Treat..

[80] Berger E, Hasking P, Martin G (2013). ‘Listen to them’: adolescents’ view on helping young people who self-injure. J Adolesc.

[81] Fadum EA, Stanley B, Rossow I, Mork E, Törmoen AJ, Mehlum L (2013). Use of health services following self-harm in urban versus suburban and rural areas: a national cross-sectional study. BMJ Open.

[82] Fortune S, Sinclair J, Hawton K (2008). Adolescents’ views on preventing self-harm: a large community study. Soc Psychiatry Psychiatr Edpidemiol..

[83] Rissanen ML, Kylma J, Laukkanen E (2009). Descriptions of help by Finnish adolescents who self-mutilate. J Child Adolesc Psychiatr Nurs..

[84] Clarke AR, Schnieden C, Hamilton BA, Dudley AM, Beard J, Einfeld SL (2004). Factors associated with treatment compliance in young people following an emergency department presentation for deliberate self-harm. Arch Suicide Res..

[85] Huey SJ, Henggeler SW, Rowland MD, Halliday-Boykins CA, Cunningham PB, Pickrel SG (2004). Multisystemic therapy effects on attempted suicide by youths presenting psychiatric emergencies. J Am Acad Child Adolesc Psychiatry.

[86] Ougrin D, Boege I, Stahl D, Banarsee R, Taylor E (2013). Randomised controlled trial of therapeutic assessment versus usual assessment in adolescents with self-harm: 2 years follow-up. Arch Dis Child.

[87] Rossouw TI, Fonagy P (2012). Mentalization-based treatment for self-harm in adolescents: a randomized controlled trial. J Am Acad Child Adolesc Psychiatry.

[88] Brent DA, Emslie GJ, Clarke GN, Asarnow J, Ritz L, Vitiello B (2009). Predictor of spontaneous and systematically assessed suicidal adverse events in the treatment of SSRI-resistant depression in adolescents (TORDIA) study. Am J Psychiatry.

[89] Taylor LMW, Oldershaw A, Richards C, Davidson K, Schmidt U, Simic M (2011). Development and pilot evaluation of a manualized cognitive-behavioural treatment package for adolescent self-harm. Behav Cogn Psychoth..

[90] Rathus JH, Miller AL (2002). Dialectical behavior therapy adapted for suicidal adolescents. Suicide Life Threat Behav.

[91] Fleischhaker C, Böhme R, Sixt B, Brück C, Schneider C, Schulz E (2011). Dialectical behavioral therapy for adolescents (DBT-A): a clinical trial for patients with suicidal and self-injurious behavior and borderline symptoms with a one-year follow-up. Child Adolesc Psychiatry Ment Health..

[92] Geddes K, Dziurawiec S, Lee CW (2013). Dialectical behaviour therapy for the treatment of emotion dysregulation and trauma symptoms in self-injurious and suicidal adolescent females: a pilot programme within community-based child and adolescent mental health service. Psychiatry J..

[93] Mehlum L, Tørmoen AJ, Ramberg M, Haga E, Diep LM, Laberg S (2014). Dialectical behavior therapy for adolescents with repeated suicidal and self-harming behavior: a randomized trial. J Am Acad Child Adolesc Psychiatry.

[94] Tørmoen AJ, Grøholt B, Haga E, Brager-Larsen A, Miller A, Walby F (2014). Feasibility of dialectical behavior therapy with suicidal and self-harming adolescents with multi-problems: training, adherence, and retention. Arch Suicide Res.

[95] Woodberry KA, Popenoe EJ (2008). Implementing dialectical behavior therapy with adolescents and their families in a community outpatient clinic. Cogn Behav Pract.

[96] Pineda J, Dadds MR (2013). Family intervention for adolescents with suicidal behavior: A randomized controlled trail and mediation analysis. J Am Acad Child Adolesc Psychiatry.

[97] Tambourou JW, Gregg E (2002). Impact of an empowerment-based parent education program on the reduction of youth suicide risk factors. J Adolesc Health.

[98] Power L, Morgan S, Byrne S, Boylan C, Carthy A, Crowley S (2009). A pilot study evaluating a support programme for parents of young people with suicidal behaviour. Child Adolesc Psychiatry Ment Health..

[99] Rissanen ML, Kylma JPO, Laukkanen ER (2008). Parental conceptions of self-mutilation among Finnish adolescents. J Psychiatr Ment Health Nurs.

[100] Morgan S, Rickard E, Noone M, Boylan C, Carthy A, Crowley S (2013). Parents of young people with self-harm or suicidal behaviour who seek help—a psychosocial profile. Child Adolesc Psychiatry Ment Health..

[101] Cassidy C, McNicholas F, Lennon R, Tobin B, Doherty M, Adamson N (2009). Deliberate self-harm (DSH): a follow-up study of Irish children. Ir Med J.

[102] Plantin L, Daneback K (2009). Parenthood, information and support on the internet: a literature review of research on parents and professionals online. BMC Family Pract.

[103] Tuffrey C, Finlay F (2002). Use of the internet by parents of peadiatric outpatients. Arch Dis Child.

[104] Wainstein BK, Sterling-Levis K, Baker SA, Taitz J, Brydon M (2006). Use of the internet by parents of paediatric patients. J Paediatr Child Health.

[105] Oh E, Jorm AF, Wright A (2009). Perceived helpfulness of websites for mental health information. Soc Psychiatry Psychiatry Epidemiol.

[106] Scharer K (2005). Internet social support for parents: the state of science. J Child Adolesc Psychiatr Nurs..

[107] Lewis SP, Mahdy JC, Michal NJ, Arbuthnott AE (2014). Googling self-injury: the state of health information obtained through online searches for self-injury. JAMA Pediatr.

[108] Self-Injury Outreach and Support (2015) http://www.sioutreach.org. Accessed 11 March 2015

[109] The Cornell Research Program on Self-Injury and Recovery: Resource (2015) http://www.selfinjury.bctr.cornell.edu/resources.html. Accessed 11 March 2015

